# Evaluation of simultaneous multi-slice acquisition with advanced processing for free-breathing diffusion-weighted imaging in patients with liver metastasis

**DOI:** 10.1007/s00330-023-10234-w

**Published:** 2023-09-30

**Authors:** Mihaela Rata, Katja N. De Paepe, Matthew R. Orton, Francesca Castagnoli, James d’Arcy, Jessica M. Winfield, Julie Hughes, Alto Stemmer, Marcel Dominik Nickel, Dow-Mu Koh

**Affiliations:** 1 Department of Radiology, MRI Unit, The Royal Marsden NHS Foundation Trust, London, UK; 2 Division of Radiotherapy and Imaging, The Institute of Cancer Research, London, UK; 3 Siemens Healthcare GmbH, MR Application Predevelopment, Erlangen, Germany

**Keywords:** Diffusion MRI, Liver, Metastasis, Patients, Simultaneous multi-slice acquisition

## Abstract

**Objectives:**

Diffusion-weighted imaging (DWI) with simultaneous multi-slice (SMS) acquisition and advanced processing can accelerate acquisition time and improve MR image quality. This study evaluated the image quality and apparent diffusion coefficient (ADC) measurements of free-breathing DWI acquired from patients with liver metastases using a prototype SMS-DWI acquisition (with/without an advanced processing option) and conventional DWI.

**Methods:**

Four DWI schemes were compared in a pilot 5-patient cohort; three DWI schemes were further assessed in a 24-patient cohort. Two readers scored image quality of all *b*-value images and ADC maps across the three methods. ADC measurements were performed, for all three methods, in left and right liver parenchyma, spleen, and liver metastases. The Friedman non-parametric test (post-hoc Wilcoxon test with Bonferroni correction) was used to compare image quality scoring; *t*-test was used for ADC comparisons.

**Results:**

SMS-DWI was faster (by 24%) than conventional DWI. Both readers scored the SMS-DWI with advanced processing as having the best image quality for highest *b*-value images (b750) and ADC maps; Cohen’s kappa inter-reader agreement was 0.6 for b750 image and 0.56 for ADC maps. The prototype SMS-DWI sequence with advanced processing allowed a better visualization of the left lobe of the liver. ADC measured in liver parenchyma, spleen, and liver metastases using the SMS-DWI with advanced processing option showed lower values than those derived from the SMS-DWI method alone (*t*-test, *p* < 0.0001; *p* < 0.0001; *p* = 0.002).

**Conclusions:**

Free-breathing SMS-DWI with advanced processing was faster and demonstrated better image quality versus a conventional DWI protocol in liver patients.

**Clinical relevance statement:**

Free-breathing simultaneous multi-slice- diffusion-weighted imaging (DWI) with advanced processing was faster and demonstrated better image quality versus a conventional DWI protocol in liver patients.

**Key Points:**

*• Diffusion-weighted imaging (DWI) with simultaneous multi-slice (SMS) can accelerate acquisition time and improve image quality.*

*• Apparent diffusion coefficients (ADC) measured in liver parenchyma, spleen, and liver metastases using the simultaneous multi-slice DWI with advanced processing were significantly lower than those derived from the simultaneous multi-slice DWI method alone.*

*• Simultaneous multi-slice DWI sequence with inline advanced processing was faster and demonstrated better image quality in liver patients.*

## Introduction

Diffusion-weighted imaging (DWI) is a valuable MRI technique widely used in oncology [1] that can detect/characterize liver tumor, assess tumor response to therapy, and predict patient outcome [2, 3].

DWI is commonly implemented on clinical MR systems as a single-shot spin-echo echo-planar technique. In the liver, DWI can be adversely affected by respiratory and cardiac motion. Three methods may be employed to mitigate motion effects on liver DWI acquisition: breath-hold, respiratory triggering, or free-breathing. Free-breathing DWI (with increased averaging per *b*-value) is usually favored [4, 5], as it results in higher signal-to-noise ratio (SNR) of DW images vs. breath-hold DWI [5] and has better reproducibility/repeatability of apparent diffusion coefficient (ADC) compared to the two other methods [6, 7]. However, a free-breathing acquisition can be long (~ 5 min) [8].

To shorten its acquisition time, free-breathing DWI can be combined with a simultaneous multi-slice (SMS) technique [9, 10] that allows data acquisition acceleration. In addition, advanced processing steps [11] can be integrated with the SMS acquisition to improve data quality. To date, free-breathing SMS-DWI with advanced processing has been reported in two studies. In the first study, SMS-DWI improved both image resolution and lesion detection/delineation in 15 adult patients with liver metastasis from neuroendocrine tumors [12]. In the second, SMS-DWI was faster than standard DWI, and demonstrated overall improvement in image quality in 16 pediatric patients with cystic fibrosis who underwent abdominal imaging [13]. However, it is unclear to what extent the advanced imaging processing added benefit to the resultant image quality compared with SMS-DWI implementation on its own.

Hence, the two aims of this study were: (1) to compare image quality obtained using conventional DWI vs. prototype SMS-DWI (with/without advanced processing) strategies in oncological patients who underwent liver MRI; and (2) to compare, within the same patient cohort, ADC values derived from the prototype SMS-DWI with/without advanced processing.

## Materials and methods

### Patients

This prospective study was approved by the local institutional review board (written informed consent from each participant was waived). Two cohorts of patients referred for clinical liver MRI between November 2018 and June 2019 were scanned. A pilot cohort consisting of 5 patients (3 females, 2 males, mean age 61 years) was used to assess four initial DWI schemes. After removal of one DWI scheme demonstrating suboptimal images, only three DWI schemes were assessed further on a main cohort of 24 patients (18 males, 6 females, mean age 63, range 36–84 years). These 24 patients had primary cancers derived from the gastrointestinal tract: colon (11), colorectal (5), rectum (4), rectosigmoid (1), anus (1), bowel (1), and unspecified (1). Liver metastases were present in 9/24 patients.

### Test object

The same four DWI schemes were tested on an ice-water phantom [14] to assess various ADC values across 5 vials, at a set temperature of 0 °C. Relative errors versus the known literature values [14] were reported.

### MRI protocol

All patients were scanned on a 1.5 T scanner (MAGNETOM Aera, Siemens Healthcare) using an anterior 18-element body-array coil combined with a posterior 32-element spine coil. All DWIs were acquired axially, in free breathing, using a 3-direction trace-weighted diffusion encoding with three *b*-values (0, 100, 750 s/mm^2^), four averages per *b*-value, and a reconstructed resolution of 1.5 × 1.5 × 6 mm^3^.

The four initial DWI series, with matched volumetric resolution and *b*-values, were: (1) conventional DWI (bipolar diffusion-encoding scheme), (2) conventional DWI (monopolar diffusion-encoding scheme), (3) prototype SMS-DWI (monopolar scheme), and (4) prototype SMS-DWI (monopolar scheme with advanced processing). MR parameters are described in Table 1. The conventional DWI was acquired with a TR = 7 s (the minimal possible TR for the bipolar sequence) and an acquisition time of 3:37 min. The SMS-DWI used a reduced TR = 5 s and a slice acceleration factor of 2 allowing faster acquisitions (2:46 min).

**Table 1:**
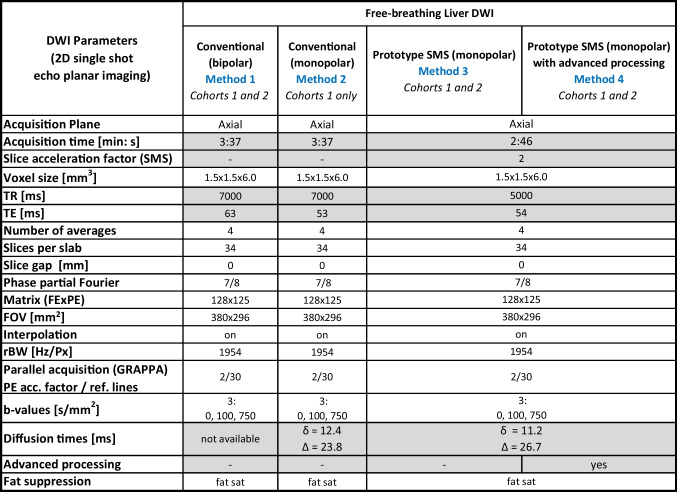
MR parameters of all four DWI sequences/three acquisitions. MR parameters that were different between the three DWI acquisitions are highlighted in grey

The monopolar conventional scheme (method 2) employs a Stejskal-Tanner type of single-refocused diffusion-sensitizing gradient [15], while the bipolar conventional scheme (method 1) uses a double-refocused diffusion-sensitizing scheme [16] which is less prone to eddy-current-induced distortions. The prototype SMS-DWI sequence was based on a monopolar diffusion-encoding scheme and automatically generated two DWI outputs from the same single acquisition: one reconstruction without advanced processing (method 3) and one reconstruction with advanced processing (method 4). Bipolar-based SMS-DWI was not investigated within the patient cohort because no advantages were found over monopolar SMS-DWI. Both bipolar and monopolar SMS-DWI were tested at TR = 5 s (preliminary test in a healthy volunteer, data not presented here) and offered the same reduction of the acquisition time. However, the longer TE needed for the bipolar SMS-DWI (75 ms vs. 54 ms for the monopolar SMS-DWI) resulted in a lower SNR and poorer image quality. Overall, the prototype SMS-DWI allowed a minimal repetition time (TR) of ~ 3 s, hence shorter acquisition, but image quality decreased with shorter TR. Therefore, for this study, a TR = 5 s was considered as a trade-off between image quality and accelerated acquisition time. TR of 5 s (~ respiratory period) may help reduce partial volume artifact in the cranio-caudal direction as well.

After the assessment of the preliminary 5-patient cohort data, the monopolar conventional DWI (method 2) was eliminated due to poorer image quality. The remaining 3 DWI methods were assessed on a further cohort of 24 patients. For each patient, a total acquisition time of < 7 min was added to their clinical protocol (~ 20% additional time). These 7 min included two DWI acquisitions (the conventional DWI and the prototype SMS-DWI) resulting in three DWI series for evaluation. The conventional DWI was based on our clinical protocol and was considered the standard clinical reference for image quality assessment. Both DWIs were acquired at the end of a contrast-enhanced clinical protocol, during the hepatobiliary phase.

The DW images and ADC maps with advanced processing were automatically generated by the scanner and implemented as part of the inline image reconstruction of the prototype SMS-DWI sequence (in addition to their non-advanced-processed counterparts). The advanced processing steps included: (1) a non-rigid 2D motion correction algorithm for images with the same *b*-value and multiple averages, (2) registration of images with different *b*-values going from lower to higher *b*-values, (3) adaptive averaging of complex-valued images before magnitude extraction of the raw images [17], and (4) intensity correction for locally corrupted images [18]. Before the calculation of the ADC maps, the combined images with different *b*-values were furthermore aligned using the same non-rigid motion correction algorithm. A detailed explanation of these processing steps is presented in [13].

### Image quality assessment

The overall image quality of each of the three *b*-value images and ADC maps for all three DWI schemes in the 24-patient cohort were assessed independently by two radiologists with > 5 and > 20 years of experience in abdominal MRI (total of 12 assessments per patient). The two reviewers were blinded to the DWI sequence information and scored the images on a 3-point Likert scale (1 = poor, 2 = fair, 3 = good quality). The overall scoring of image quality assessed the liver contour sharpness, tumor/vasculature conspicuity, intra-/inter-slice signal homogeneity, image distortion, and image sharpness. As not all patients had liver metastases, the sequences were not assessed specifically for metastatic disease identification. Furthermore, a previous study [12] has reported the potential value of SMS-DWI for improving liver metastasis detection.

### ADC quantitative assessment

**Fig. 1:**
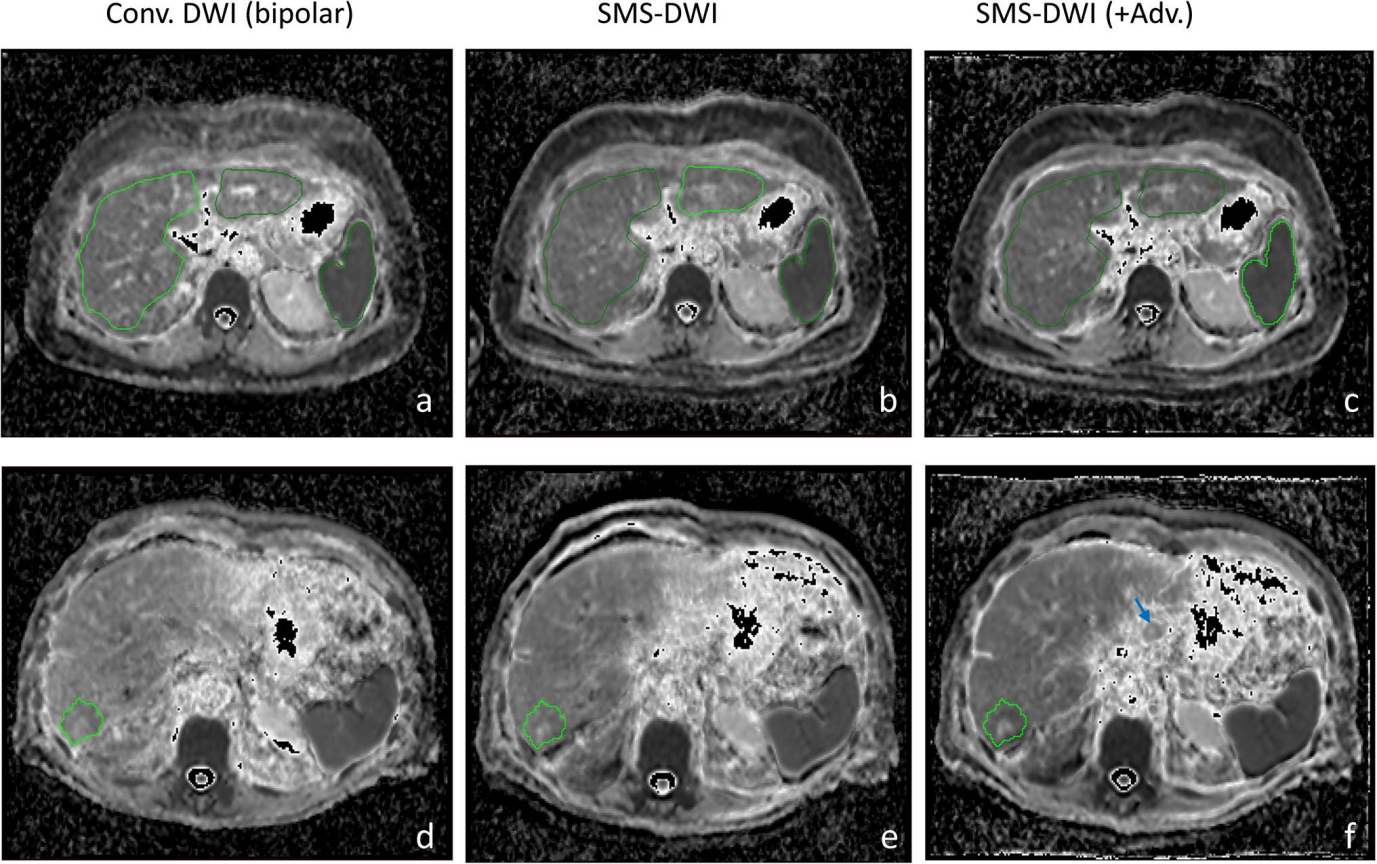
Examples of ROI overlayed on ADC maps (using 3 *b*-values) for quantitative assessment; cohort 2 (24 patients). Conv. = conventional; Adv. = advanced processing. **a**–**c** ROIs of normal areas (left liver, right liver, and spleen) obtained from a 47-year-old woman with colon cancer. ROIs specific to right liver (**a**), left liver (**b**), and spleen (**c**) are highlighted within each panel. **d**–**f** Metastatic lesion ROI within the liver of a 64-year-old man with rectal cancer; note an additional small lesion (blue arrow) clearly visible on the prototype SMS-DWI with advanced processing

Regions of interest (ROIs) were manually drawn over normal-appearing liver on a central slice of the *b* = 750 s/mm^2^ series (b750) associated with the 3 DWIs (conventional bipolar, prototype SMS, and prototype SMS with advanced processing) and then copied to the matched ADC map in each patient. Caution was considered to avoid the inclusion of very big vessels which may further impact ADC measurements. The central slice was chosen to ensure that, within the same slice, ROIs could be drawn in the right and left liver lobes and spleen (see Fig. 1a–c).

Additionally, ROIs were defined for liver metastases (where present), and the entire tumor was delineated, including any necrotic regions (Fig. 1d–f). To include sufficient voxels for reliable ADC measurement, metastases with a diameter > 2 cm were considered. ROI-based ADC estimates for the four sites (left liver, right liver, spleen, and liver lesion) were calculated for each DWI scheme across all 24 patients. ADC maps were calculated twice: (1) using all acquired three *b*-values (0, 100, and 750 s/mm^2^); (2) using only the highest two *b*-values (100 and 750 s/mm^2^) to minimize the impact of perfusion on ADC calculations.

Statistically, only the ADC measurements resulting from the prototype SMS-DWI method (with/without advanced processing) were compared. A statistical comparison with the conventional DWI was not considered suitable because of the differences in the diffusion-encoding scheme (bipolar for conventional DWI vs. monopolar for prototype SMS-DWI).

### Statistics

#### Image quality

Image quality scores from each observer were summarized as means for each of the 12 assessments (4 images across 3 DWI methods). The Friedman non-parametric tests (MATLAB, R2019a, MathWorks) compared the image scoring for the 3 DWI approaches. Where the Friedman results were significant, further post-hoc analysis based on the pairwise Wilcoxon tests (signed rank) with the Bonferroni correction was performed. The inter-observer agreement was assessed using kappa statistics (quadratic weighting) with kappa values of 0.01–0.20 representing slight agreement, 0.21–0.40 fair, 0.41–0.60 moderate, 0.61–0.80 substantial, and 0.81–1.00 excellent [19].

#### ADC measurements

Means and standard deviations of cohort ADC (using three and two *b*-values) for each DWI method and each anatomical location were reported. A *t*-test was used to assess whether ADC estimates from SMS-DWI with advanced processing were significantly different from SMS-DWI without advanced processing.

## Results

### Test object

**Fig. 2:**
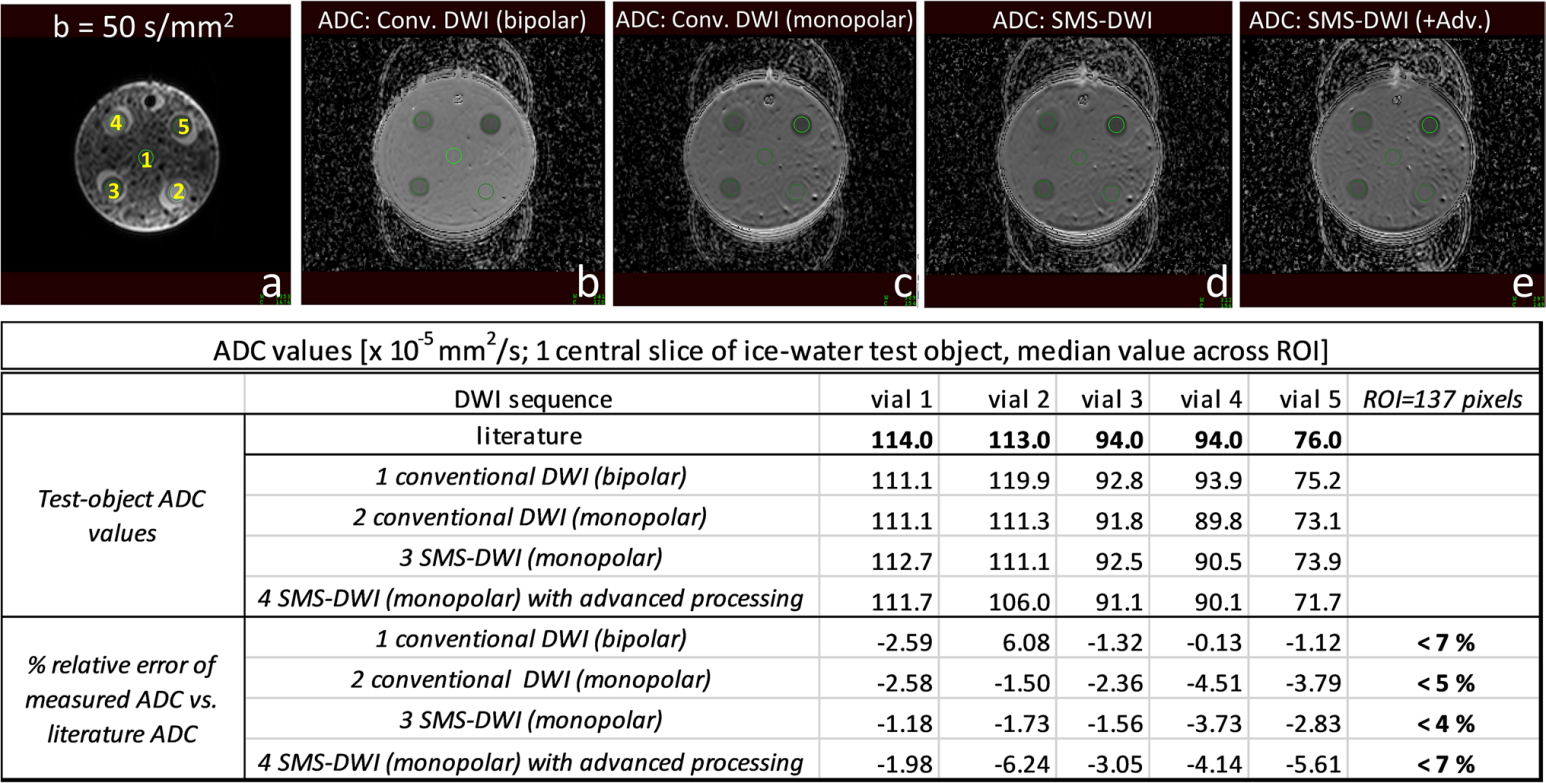
ADC results derived from the test-object experiment. All 3 *b*-values were used for the ADC maps calculation in this context. *Conv*. = conventional; *Adv*. = advanced processing

ADC estimates (using all three *b*-values) and their relative errors are summarized in Fig. 2. The ADC relative error across all 5 vials was between − 6.24 and 6.08%. Similar ADC ranges were observed for all four methods.

### Image quality

**Fig. 3:**
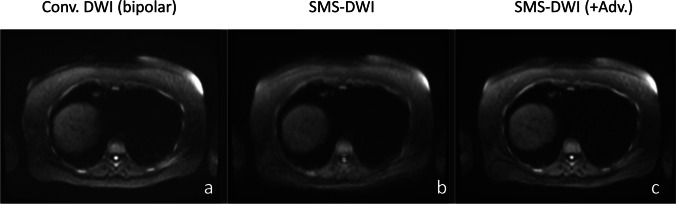
Examples of high *b*-value images (*b* = 750 s/mm^2^) from a 51-year-old woman, acquired with conventional DWI (**a**), SMS-DWI (**b**), and SMS-DWI with advanced processing (**c**). In panel **c**, a better delineation of the liver dome can be observed when comparing to panel **b**

**Fig. 4:**
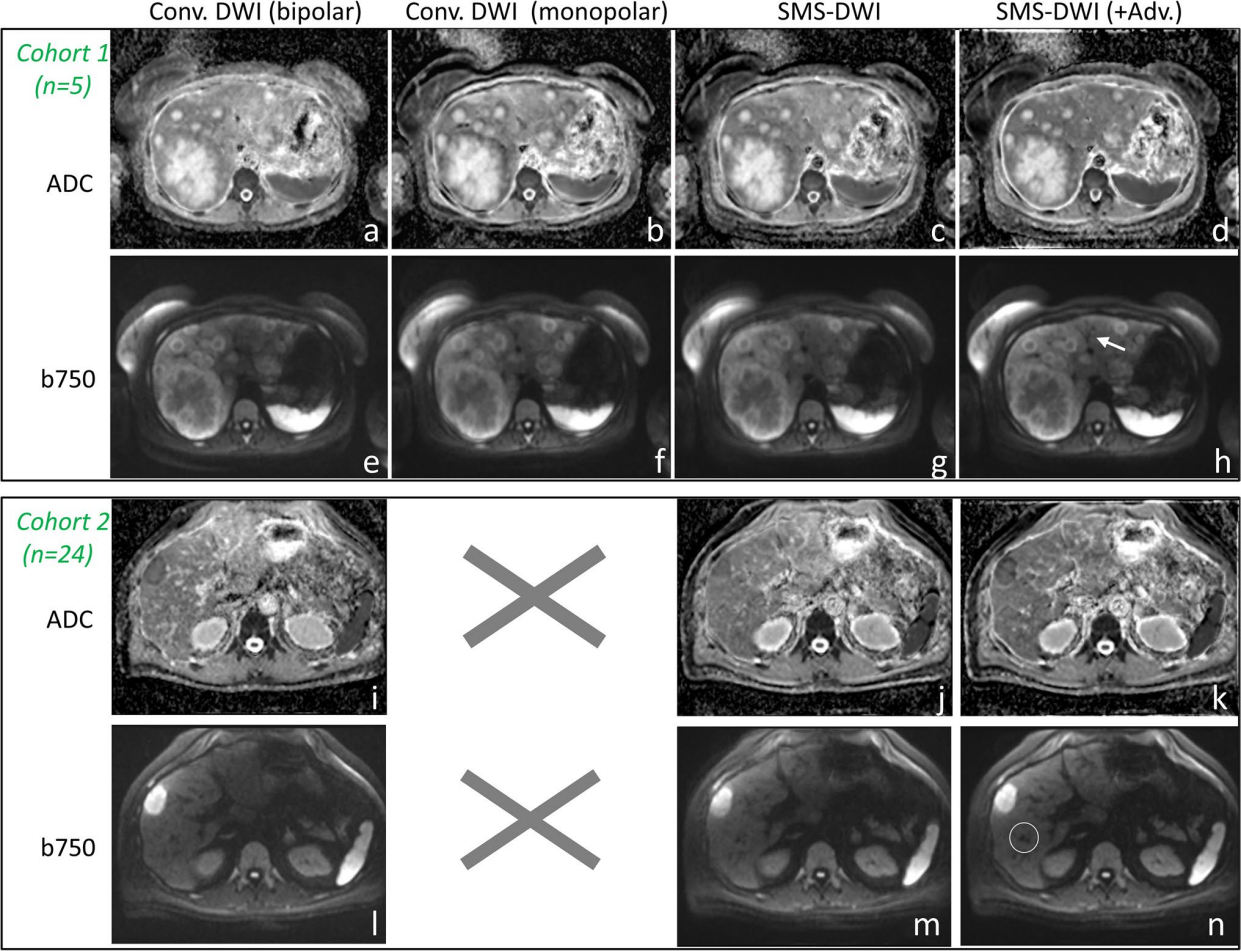
Examples of high *b*-value images (*b* = 750 s/mm^2^) and ADC maps (derived from 3 *b*-values) of liver metastases from two patients from each cohort: a 53-year-old woman (top/cohort 1) with colorectal cancer and a 61-year-old man with bowel cancer (bottom/cohort 2). Note the improved signal homogeneity across the slice on both *b*-value image and ADC map when using the prototype SMS-DWI with advanced processing (columns 1 vs. 4). Moreover, the advanced processing allows for a better delineation of the liver or the blood vessels as seen on the *b*-value images (white arrow and circle in column 4). *Conv*. = conventional; *Adv*. = advanced processing

The conventional DWI acquisition time (3:37 min) was reduced by 24% when using the SMS-DWI (2:46 min). Figure 3 shows b750 images for conventional and SMS-DWI for a slice located near the liver dome in an area most affected by motion. Examples of b750 images and ADC maps from two patients with liver metastases from colorectal (rows 1 and 2) and bowel carcinoma (rows 3 and 4) are presented in Fig. 4. Columns 1 and 2 display images derived from conventional DWI, while the SMS-DWI results are shown in columns 3 and 4.

The prototype SMS-DWI with advanced processing demonstrates a more homogeneous signal across the slice, e.g. the left liver lobe (column 4 vs. 1, Fig. 4). The advanced processing reduced motion artifacts and generated crisper images with improved conspicuity of the liver (Figs. 3, 4) or blood vessels (see white arrow on panel h or white circle on panel n on Fig. 4). Similarly, a small lesion is clearer visible on the SMS-DWI method with advanced processing (see blue arrow on Fig. 1f).

The summary of all radiological scoring across the 24-patient cohort is shown in Table 2. The prototype SMS protocol with advanced processing was scored the highest in terms of image quality by both observers for b100, b750, and ADC. The SMS-DWI sequence without advanced processing was rated the worst for the b750 image evaluation.

**Table 2:** Mean scores of the overall image quality for each type of image (b0, b100, b750, and ADC map) and each DWI method as derived from cohort 2 (24 patients). The series with the highest score for each image type is shown in bold for each observer. Images were scored from 1 (lowest) to 3 (highest). *Conv*. = conventional, *Adv*. = advanced processing

Mean scores (image quality)	b0			b100			b750			ADC		
	Conv. DWI	SMS-DWI	SMS-DWI (+ Adv.)	Conv. DWI	SMS-DWI	SMS-DWI (+ Adv.)	Conv. DWI	SMS-DWI	SMS-DWI (+ Adv.)	Conv. DWI	SMS-DWI	SMS-DWI (+ Adv.)
Observer 1	**3.00**	2.79	2.88	2.83	2.67	**3.00**	2	1.67	**2.83**	1.92	1.79	**2.79**
Observer 2	1.71	2.33	**2.71**	1.46	1.88	**2.96**	1.63	1.38	**3.00**	1.04	1.96	**3.00**

**Table 3:** Statistical test results demonstrating the preference of the two readers of the SMS with advanced processing DWI method over the conventional and SMS methods. Note that in order to account for the Bonferroni correction, the obtained *p* values were multiplied by 3 (as three methods were compared), and a *p* value lower than 0.05 was considered statistically significant. All values statistically significant (*p* < 0.05) are bolded. *Conv*. = conventional, *Adv*. = advanced processing

Statistics: *p* values		b0	b100	b750	ADC
Observer 1	*Friedman test *	**0.02**	**0.01**	** < 0.0001**	** < 0.0001**
	Post -hoc* tests (Wilcoxon signed rank* + *Bonferroni):*				
	Conv. DWI (bipolar) vs. SMS-DWI	0.19	1.03	0.29	1.59
	SMS-DWI vs. SMS-DWI (+ Adv.)	1.00	**0.02**	** < 0.001**	** < 0.0001**
	Conv. DWI (bipolar) vs. SMS-DWI (+ Adv.)	0.75	0.38	** < 0.001**	** < 0.001**
Observer 2	* Friedman test*	** < 0.0001**	** < 0.0001**	** < 0.0001**	** < 0.0001**
	Post-hoc* tests (Wilcoxon signed rank* + *Bonferroni):*				
	Conv. DWI (bipolar) vs. SMS-DWI	**0.002**	0.10	0.66	** < 0.0001**
	SMS-DWI vs. SMS-DWI (+ Adv.)	0.14	** < 0.0001**	** < 0.0001**	** < 0.0001**
	Conv. DWI (bipolar) vs. SMS-DWI (+ Adv.)	**0.002**	** < 0.0001**	** < 0.0001**	** < 0.0001**

The Cohen kappa test statistics found moderate agreement between the readers when assessing ADC maps (*k* = 0.56, *p* < 0.0001) and b750 images (0.60, *p* < 0.0001). The Friedman tests were statistically significant across all DWI approaches. Results of the further post-hoc analysis are presented in Table 3, confirming again the superiority of the prototype SMS-DWI with advanced processing method against the other two sequences.

### ADC measurements

**Fig. 5:**
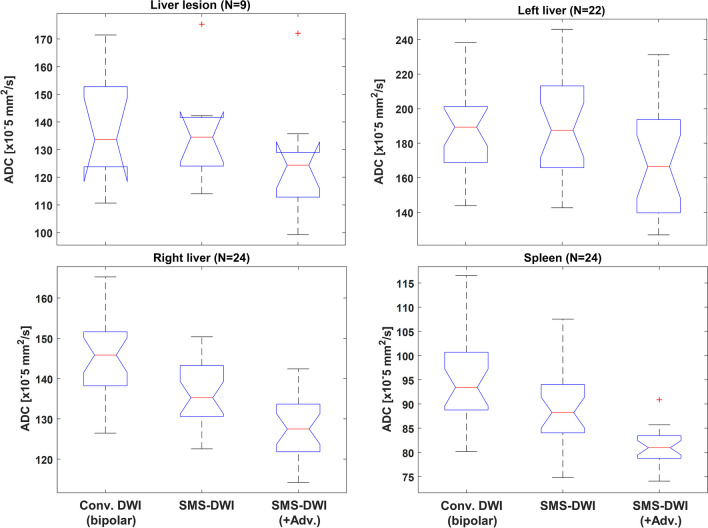
Boxplots of patient cohort ADC estimates in liver lesion and three anatomical locations (left liver, right liver, spleen) obtained using three DWI methods. ADC was calculated using all 3 *b*-values. Conv. = conventional; Adv. = advanced processing. Boxplot legend: red central line = median; blue line limits = 25% and 75% percentiles; black line limits = most extreme datapoints that are not outliers; red cross = outlier. Notches are introduced to ease visual comparison between groups: non-overlapping notches suggest statistically significant different medians between groups. Note that the inverted notches for the liver lesion panel suggest a larger confidence interval for the median value (than the 1st quartile) due to a smaller sample size for this subcohort

Two out of 24 patients did not have a left liver lobe following surgery, and only 9/24 patients had measurable liver metastases; therefore, smaller cohorts were considered for these two locations (22; 9 patients). A complete cohort of 24 patients was considered for the right liver lobe and spleen. Boxplots of cohort ADC measurements (using all three *b*-values) for each DWI method and for each anatomical location are presented in Fig. 5. ADC estimates from SMS-DWI with advanced processing were significantly lower than ADC estimates from SMS-DWI without advanced processing. This observation was consistent for each of the four anatomical locations and was maintained for the ADC measurements derived from the two-*b*-value analysis. The mean and standard deviations of ADC are presented in Table 4, together with *p* values of the *t*-tests.

**Table 4:** Summary of ADC measurements (mean and standard deviation, SD) using the three DWI methods across all 24 patients. *t*-test compared results from SMS-DWI vs. SMS-DWI with advanced processing. *Conv.* = conventional, *Adv.* = advanced processing

ADC [× 10^–5^ s/mm^2^]			Conventional DWI (bipolar)	SMS-DWI	SMS-DWI (+ Adv.)	*p* values of *t* test
3-*b*-value analysis	Left liver	Mean	186.4	192.3	169.6	< 0.0001
		SD	23.3	30.0	32.8	-
	Right liver	Mean	145.8	136.9	128.1	< 0.0001
		SD	9.9	8.3	7.9	-
	Spleen	Mean	95.4	89.6	81.3	< 0.0001
		SD	9.3	8.3	3.7	-
	Lesion	Mean	137.5	135.7	124.3	0.002
		SD	19.1	17.9	21.1	-
2-*b*-value analysis	Left liver	Mean	154.40	167.18	149.00	< 0.0001
		SD	22.8	27.9	31.3	-
	Right liver	Mean	123.30	116.54	109.06	< 0.0001
		SD	8.5	7.9	6.7	-
	Spleen	Mean	88.40	83.43	75.22	< 0.001
		SD	9.4	8.5	4.1	-
	Lesion	Mean	132.8	126.8	118.2	0.009
		SD	20.2	19.0	21.7	-

## Discussion

### Qualitative assessment

This study demonstrates the qualitative superiority of a prototype SMS-DWI method with advanced processing over SMS-DWI alone or conventional DWI in the liver. SMS-DWI without advanced processing failed to perform better than the conventional DWI method (with bipolar diffusion-encoding scheme). Our implemented SMS-DWI method was based on a monopolar acquisition, which could explain its lower scoring for the b750 image without advanced processing. The higher *b*-value results in stronger eddy-current-induced distortions between different diffusion directions that are reduced by bipolar gradients or advanced processing. In this study, we did not consider a bipolar-based prototype SMS-DWI sequence, as initial tests at sequence setup did not accelerate acquisition or improve image quality compared to its equivalent monopolar SMS-DWI.

These qualitative results agree with two other studies that have investigated the prototype SMS-DWI method with advanced processing in patients with metastatic liver [12] or cystic fibrosis [13]. Similarly to [18], the image quality improvement was particularly noticeable for the left liver lobe, which is usually affected by cardiac motion. Improved visualization of the left liver lobe could help in better discernment for smaller metastatic lesions (see Fig. 1f, blue arrow) and therefore impact the treatment plan. Note that the advanced processing included techniques for the removal of corrupted signal data from cardiac pulsation and in-plane registration of the images, but this did not account for effects in the cranio-caudal direction (the most affected by motion as demonstrated in [20]).

### Quantitative assessment

Our implementation of SMS-DWI resulted in a 24% decrease in acquisition time (vs. the conventional DWI), which can help towards increasing throughput in busy radiological departments. Otherwise, for the same acquisition time, the thickness of the slice can be halved (as demonstrated in [12]) and smaller lesions, less affected by partial volume effect, could be then detected.

The bipolar conventional DWI used in this comparative study was considered the standard clinical reference for qualitative assessment purposes only. Specific parameters for the bipolar conventional DWI method such as different diffusion times, longer TE, and TR may affect its ADC estimates [21]. Therefore, the conventional DWI (bipolar) was not included in the quantitative comparison of ADCs. All ADCs derived from the three methods were reported, but only the SMS-DWI vs. SMS-DWI with advanced processing comparison was followed up with statistical tests. Such *t*-tests found a highly statistically significant difference in ADC means between the two SMS-DWI methods and across all four abdominal locations. ADC estimated using the prototype SMS-DWI with advanced processing option was consistently lower than the corresponding ADC from non-corrected SMS-DWI which was observed in patients and a test object. A lower ADC when performing the same advanced processing was recently reported [22], although the assessment was associated with a standard DWI only (without SMS acceleration). Considering the intertwined effects of the main features of advanced processing (registration across averages and *b*-values, complex-averaging and local signal drop correction), it is difficult to explain the lower ADC values without running a dedicated study investigating the separate contribution of each feature. A correction generating lower ADC values by postprocessing methods that aim to address local signal loss was reported in a recent work [23] and is consistent with our findings. Various other methods for prospective correction of signal loss are currently under research [24, 25].

To disentangle the contributions of pseudo-perfusion and motion on ADC estimation, two ADC analyses were performed (two vs. three *b*-values). As expected, across all four anatomical locations, all two *b*-value ADCs (without *b* = 0 s/mm^2^) were lower than their corresponding three *b*-value ADCs. The same ranking order of ADC values per method was demonstrated in both analyses at all four locations. For 3/4 locations (right liver, spleen, and metastases), ADC was highest for conventional DWI, intermediate for SMS-DWI, and lowest for SMS-DWI with advanced processing. For the left liver lobe, the highest ADC was measured by SMS alone suggesting the higher sensitivity to motion for the monopolar-based SMS method.

Although not directly comparable, our results agree with previous reports confirming lower ADC estimates in the liver when using prototype SMS-DWI vs. conventional DWI [9, 10]. Both studies had a shorter TR (2400 ms; 1156 ms) vs. the TR of the conventional DWI and explained the lower ADC by a T_1_ saturation effect in cases of lower TR. We used a much longer TR for the SMS-DWI sequence (5000 ms), which should allow a sufficient recovery of the T_1_ signal in the liver (> > 5 times of reported T_1_ of the liver [26]), hence a minimal T_1_ saturation effect on the derived ADC. Nevertheless, we still observed a lower ADC for monopolar SMS-DWI (vs. bipolar conventional DWI). We hypothesize that ADC estimates might have been impacted by the type of sensitizing gradient schemes used in our study. Monopolar ADC is expected to be significantly lower than bipolar ADC [27], and such behavior was confirmed in our study.

We reported ADC quantification based on a prototype SMS-DWI with advanced processing for four different locations. Glutig [13] used the same method and reported ADC (from 3 *b*-values, no b0 data) of 1.12 × 10^−3^mm^2^/s for segment 5 of liver, 1.19 × 10^−3^mm^2^/s for segment 4, and 0.8 × 10^−3^mm^2^/s for spleen that are consistent with our ADC of 1.09 × 10^−3^mm^2^/s for the right liver and 0.75 × 10^−3^mm^2^/s for the spleen (the two-*b*-value analysis, no b0 data). Chen [6] showed that ROI-derived ADC in the liver decreases from left to right, following the degree of motion impacting the liver that also decreases from left to right. Our ADCs were derived from a ROI covering the whole right liver (i.e., averaging over other liver segments less impacted by motion), so an overall slightly lower ADC compared with values from reference [13] may be expected. Additionally, ADC of the right liver lobe reported by Xu [12] using the identical prototype SMS method was very similar to our values (1.05 vs. 1.09 × 10^−3^mm^2^/s).

### Limitations

To our knowledge, there are no reports on ADC measurements using a prototype SMS-DWI method with advanced processing in metastatic liver lesions, so no direct comparison with the literature was available. Only 9/24 patients had sizeable liver metastases, so the reported values are limited by this small cohort. Additionally, these patients were scanned clinically at various points during their treatment journey, so a mix of treated/untreated metastases was expected. Treated metastases displaying a necrotic core were observed in 5/9 patients; ADC of these cores might have contributed to a slight increase in the cohort ADC. Our quantitative comparative study across the 24 patients is limited by the absence of a truly identical non-SMS method (i.e., a monopolar conventional DWI) to compare with the SMS method. Nevertheless, our findings agree with previous literature [9, 10] suggesting that ADC estimates from SMS-DWI are typically lower than those derived from conventional DWI. Moreover, as demonstrated in this study, SMS-DWI with advanced processing generates motion-corrected ADC values that are even lower than their corresponding ADC derived from a non-advanced-processed SMS-DWI method, in agreement with [22]. Such important observation should be considered when reporting quantitative ADC values acquired with the prototype SMS method and advanced processing. Note, however, that the reduced ADC values observed with the SMS-DWI methods (with and without advanced processing) affect the whole image and do not impede on discrimination between normal and non-normal liver parenchyma or longitudinal follow-up.

### Conclusion

In conclusion, free-breathing liver DWI based on a prototype SMS protocol with an advanced processing method was faster and demonstrated better image quality when compared with conventional DWI. Such improved image quality is particularly important in the left lobe of the liver, which is usually affected by motion. Quantitatively, ADC measured in liver parenchyma, spleen, and metastatic liver lesions using the SMS-DWI with an advanced processing method was significantly lower than those derived from the SMS-DWI method alone.

## Abbreviations

ADC: Apparent diffusion coefficient
DWI: Diffusion-weighted imaging
ROI: Region of interest
SMS: Simultaneous multi-slice
SNR: Signal-to-noise ratio
TE: Echo time
TR: Repetition time

## Declarations

### Guarantor

The scientific guarantor of this publication is Prof. Dow-Mu Koh.

### Statistics and Biometry

No complex statistical methods were necessary for this paper.

### Methodology

• prospective

• diagnostic or prognostic study

• performed at one institution
